# Comparing the distribution of neuropsychiatric symptoms among individuals with depression and mild cognitive impairment

**DOI:** 10.1192/j.eurpsy.2023.416

**Published:** 2023-07-19

**Authors:** A. Keng, D. Kapustin, C. Ma, K. Bingham, C. Fischer, L. Mah, D. Gallagher, M. A. Butters, C. R. Bowie, A. Voineskos, A. Graff, A. Flint, N. Herrmann, B. Pollock, B. Mulsant, T. Rajji, S. Kumar, P.-M. Study Group

**Affiliations:** ^1^Psychiatry, University of Toronto; ^2^Psychiatry, Baycrest Hospital; ^3^ Centre for Addictions and Mental Health; ^4^Dalla Lana School of Public Health, University of Toronto; ^5^Psychiatry, University Health Network; ^6^Psychiatry, Unity Health, Toronto; ^7^Psychiatry, Baycrest Hospital; ^8^Psychiatry, Sunnybrook Hospital, North York; ^9^Psychiatry, University of Pittsburgh, Pittsburgh; ^10^Psychiatry, Queens University, Kingston; ^11^Psychiatry, Centre for Addictions and Mental Health, Toronto, Canada

## Abstract

**Introduction:**

Neuropsychiatric symptoms (NPS) are common during the course of neurocognitive disorders. NPS have been previously reported in early and late stages of Alzheimer’s Disease. However, our understanding of NPS in high-risk states for dementia such as mild cognitive impairment (MCI) and major depressive disorder (MDD) is poor.

**Objectives:**

To compare the frequency and factor structure of neuropsychiatric symptoms among individuals with Mild Cognitive Impairment (MCI), Major Depressive Disorder (MDD) in remission, and comorbid MCI and MDD (in remission) (MCI-D).

**Methods:**

We used baseline data from the Prevention of Alzheimer’s Dementia with Cognitive Remediation Plus Transcranial Direct Current Stimulation in Mild Cognitive Impairment and Depression (PACt-MD) study, a multicenter trial across five academic sites in Toronto, Canada (clinical trial No. NCT0238667). We used ANOVA or χ2-test to compare frequency of NPS across groups. We used factor analysis of Neuropsychiatric Inventory Questionnaire (NPI-Q) items in the three groups.

**Results:**

We included 374 participants with a mean age of 72.0 years (SD = 6.3). In the overall sample, at least one NPS was present in 64.2% participants, and 36.1 % had at least moderate severity NPS (36.1%). Depression (54%, χ^2^ < 0.001) and apathy (28.7%, χ^2^=0.002) were more prevalent in the MCI-D group as compared to MCI and MDD groups. In factor analysis, NPS grouped differently in MCI, MDD, and MCI-D groups. A “psychotic” subgroup emerged among MCI and MCI-D, but not in MDD. Night-time behaviors and disinhibition grouped differently across all three groups.

**Conclusions:**

Prevalence of NPS seems higher in persons with MCI-D as compared to those with only MCI or MDD. The factor structure of NPS differed between MCI, MDD, and MCI-D groups. Future studies should investigate the association of NPS factors with cognition, function, and illness biomarkers.

**Disclosure of Interest:**

None Declared

